# On the Progression of COVID-19 in Portugal: A Comparative Analysis of Active Cases Using Non-linear Regression

**DOI:** 10.3389/fpubh.2020.00495

**Published:** 2020-09-11

**Authors:** Ana Milhinhos, Pedro M. Costa

**Affiliations:** ^1^Instituto de Tecnologia Química e Biológica António Xavier, Universidade Nova de Lisboa, Oeiras, Portugal; ^2^Faculty of Sciences, BioISI – Biosystems & Integrative Sciences Institute, University of Lisboa, Lisbon, Portugal; ^3^UCIBIO—Research Unit on Applied Molecular Biosciences, Departamento de Ciências da Vida, Faculdade de Ciências e Tecnologia da Universidade Nova de Lisboa, Almada, Portugal

**Keywords:** coronavirus, modeling, non-linear estimation, European Union, statistical forecasting

## Abstract

Portugal is often portrayed as a relatively successful case in the control of COVID-19's March 2020 outbreak in Europe due to timely confinement measures, commonly referred to as the “lockdown”. As in other European Union member states, by late April, Portugal was preparing the phased loosening of such measures scheduled for the beginning of May. Despite a modest reduction in infection rates by that time, there was insufficient data to reliably forecast imminent scenarios. Using the South Korea data as scaffold, which became a paradigmatic case of recovery following a high number of infected people, we fitted the Portuguese data to biphasic models using non-linear regression and compared the two countries. The models, which yielded a good fit, showed that recovery would be slow, with over 50% active cases months after the lockdown. These findings acted at the time as a warning, showing that a high number of infected individuals, together with an unknown number of asymptomatic carriers, could increase the risk of a slow recovery, if not of new outbreaks. A month later, the models showed more favorable outcomes. However, shortly after, as the effects of leaving the lockdown became evident, the number of infections began rising again, leaving Portugal in a situation of inward and outward travel restrictions and baffling even the most conservative forecasts for the clearing of the pandemic.

## Introduction

The first documented case of COVID-19 infection in Portugal dates back to March 2, 2020. Motivated by the rapid progression in other countries, especially in neighboring Spain [see (1, 2)], the country moved swiftly to control dissemination by shutting down many public services and imposing strict confinement measures [see, for instance, (3)]. These restrictions date from mid-March. By mid-April, when our first models were being prepared, the Portuguese government and its competent health authorities were planning the phased cessation of the lockdown measures, in alignment with the European Union guidelines. It was then consensual that the spreading of COVID-19 in Portugal, with respect to the number of infected people, fatalities, and intensive care unit (ICU) internments, was reaching a plateau when nearing the end of the lockdown. Hence, Portugal's strategy seemed a potential case of success as, by mid-April, fatalities were kept below 1,000 and the healthcare system did not attain saturation. Our first models, made readily available as a pre-print (4), aimed at providing a cautionary tale on the risks of premature easing of confinement measures when the numbers of accounted (and unaccounted) active cases (i.e., infected people) were potentially high. It was clear then that the weeks preceding the end of lockdown were critical to know what to expect from the progression of the disease in the country and how safe it was to begin the relief of the confinement measures. However, the available data were markedly insufficient to draw solid forecasts even on a short term. At this stage, epidemiological SIR (“susceptible,” “infected,” and “recovered”) models were indeed difficult to produce in Portugal and elsewhere.

Lessons could be learned, however, from the few countries believed to be clearing the pandemic. The Republic of Korea is a key case study not just due to the overall positive progress but also because the country implemented strict confinement measures, imposed timely limitations to in-bound traveling, and closed public services, such as schools. Also, South Korea endured a high number of total infections (which offers statistical significance), albeit a relatively low mortality rate, estimated at 0.9% by mid-March, when the cases totaled almost 8,000, according to the Republic of Korea COVID-19 National Emergency Response Center (5). The basic reproduction number (*R*_0_) has still been estimated at 1.5 ± 0.1 (6), therefore within the magnitude of the influenza outbreak in 1918 (7). Altogether there are significant similarities between countries even though there are likely differences in public behavior, awareness, or susceptibility. We therefore intended to model the progression of active cases in Portugal by means of non-linear regression using the Korean data as scaffold. The current work aims primarily at comparing the progression of COVID-19 in Portugal before and after the easing of the confinement measures. This information is of particular relevance as current data (July) greatly differ from the positive indications from the preceding months due to secondary outbreaks (especially, but not exclusively, in the Lisbon area).

## Methods

We used non-linear estimation to produce four-parameter log-logistic models for cumulative data, such as the total number of infections and casualties. As the number of active cases is modulated by case clearance (either by death or full recovery), we opted for a five-parameter log-Gaussian distribution biphasic asymmetric response model, as described by Martin-Betancor et al. (8). The model parameters were obtained through least squares estimation using the package “drc” for R. All statistics were performed using R 3.5 (9). The Portuguese data were compiled from the official daily reports on confirmed infections by COVID-19 as provided by the General Directorate for Health (DGS) (available at https://covid19.min-saude.pt/ponto-de-situacao-atual-em-portugal/). The data on recovered patients divulged on May 30 were redistributed between April 25 and May 30 due to delayed public release, as reported by DGS. The data from South Korea were retrieved from Worldometer (https://www.worldometers.info/coronavirus/). All data are provided in the Supplementary Information.

## Results and Discussion

**Figure 1:**
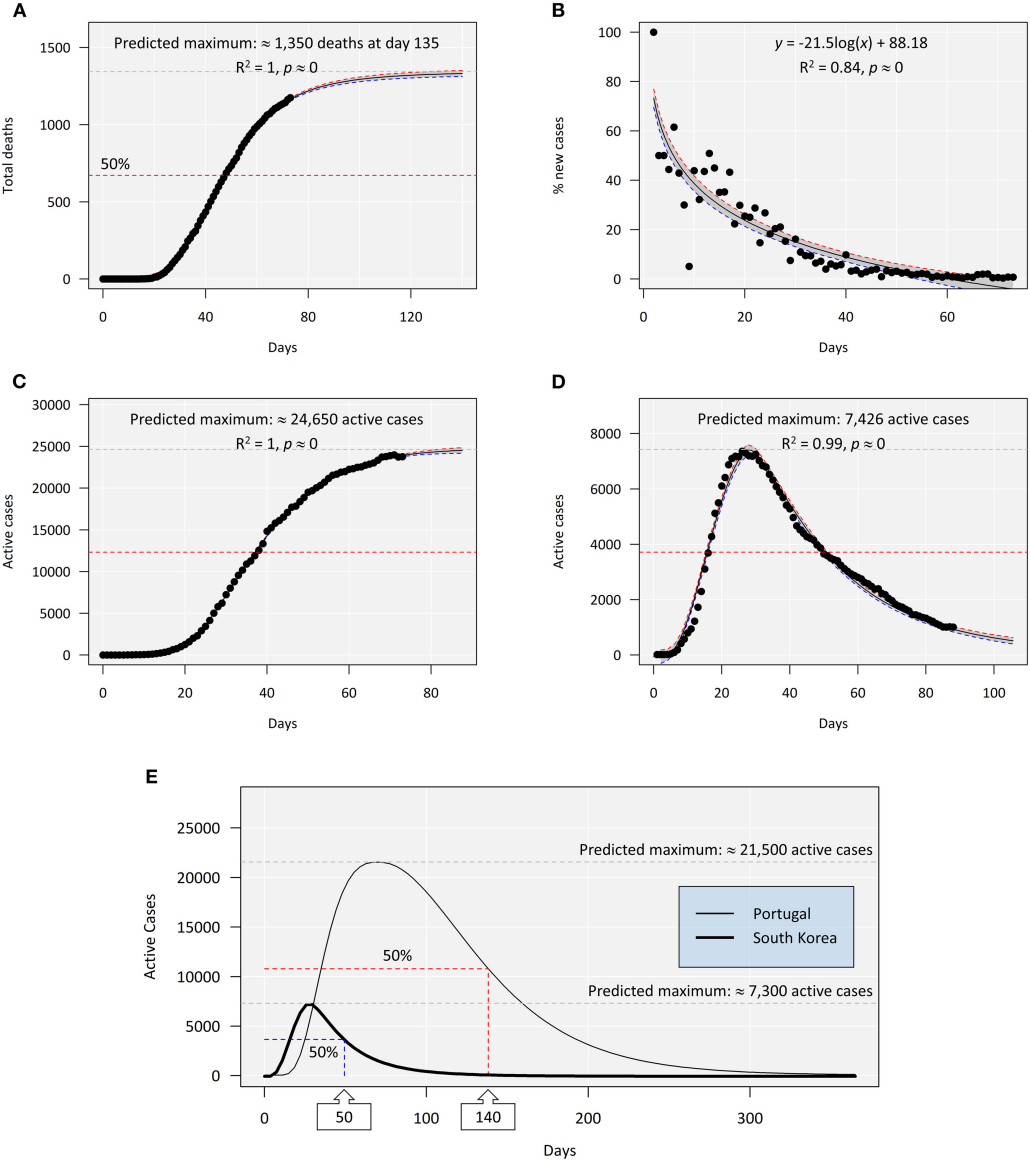
An overview of the evolution of COVID-19 in Portugal from March 1 (day 0) to April 19 (day 49), 2020, i.e., before the phased end of confinement measures. **(A)** Cumulative number of deaths fitted to a log-logistic model. The scale was extended to highlight the quality of fit and the predicted asymptotic limit (≈1,000 deaths). **(B)** A simple log-linear regression for the percentage of daily new cases (infected subjects) relative to the cumulative new cases. **(C)** Total active cases (i.e., total cases excluding deaths and recoveries) fitted to a log-Gaussian (asymmetric) model with an estimated maximum at ≈21,500 cases, highlighting the near-perfect fit to the growth phase of the model. **(D)** Active cases reported in the Republic of Korea between February 15 and April 19 fitted to a log-Gaussian model as before. The South Korean scenario already has sufficient data to fit both growth and decrease phases, again yielding a near-perfect fit. **(E)** Juxtaposition of the predicted models (scaled to a full year from the first day of reported cases) for Portuguese and Korean data (log-Gaussian non-linear regression). The models highlight the maxima and the half-maximal estimates (50% of cases recovered). Whereas South Korea already surpassed the estimate (as day 50 corresponds to April 4), in Portugal, day 140 means July 17. The shaded areas between the red and the blue dashed lines indicate 95% confidence intervals around the predicted model. The actual observations are juxtaposed to the models (∙). The *R*^2^ goodness-of-fit statistic means quadratic Spearman's rho.

The models built using data from the beginning of the first outbreak and up to April 19 (pre-lockdown easing) are summarized in Figure 1. The maximum number of infections in Portugal was then estimated to reach 25,500. The same model yielded a maximum of about 1,000 daily fatalities by day 116 (June 24), starting from day 1 (March 2) when the first cases were reported, which landmarks the beginning of the outbreak in Portugal (Figure 1A). The mortality rate was estimated at 3.5 % (as per April 19), well below that of Italy at over 10% (10). One of the most positive signs of COVID-19 control in Portugal was considered to be the reduced percentage of daily cases at the time (Figure 1B). This information must, nonetheless, be interpreted with caution because the reporting of new cases has been highly variable, in part due to increased testing during lockdown. Additionally, the recovery rates in Portugal were low at this stage (only 610 cases by April 19), which is seemingly accordant with reports from elsewhere. It must also be emphasized that, on April 19, Portugal had yet to reach the peak of active cases, which means that data could not be fitted to the descending phase of the curve, potentially biasing (likely overestimating) the model. Still the fit was nearly perfect to the ascending phase (Figure 1C). Moreover, the Korean data also fitted the same model perfectly, yielding, as expected, slower recovery than infection rates (Figure 1D). By juxtaposing the two models and expanding them to a full-year timeframe, the differences between the two countries became evident (Figure 1E). At day 50, South Korea reported 3,591 active cases, whereas the model estimated 3,653 cases (half of the 7,307 projected maximum), with the real maximum being 7293, which, again, shows the good fit of the model. Still, the model indicated that Portugal could reach 50% of recoveries only after 140 days.

The different shapes of the curves reflected in the differential parametrization of models (Table 1) should reflect not only the number of infected cases but also the different rates of recovery. Even though the Korean data validates the model, caution is mandatory when interpreting the Portuguese model as the data were incomplete and the model parameters were sure to change in time, either accelerating or slowing recovery, depending on the success of the mitigation measures and on how the loosening of confinement policies, projected to begin in May, would proceed. It was clear, though, that recovery would be long. With 50% cases still active by July, the risks of new peaks were high, furthermore considering the high percentage of untraced asymptomatic carriers of COVID-19 (11).

**Table 1:** Summary of parameter estimates for the fitting of active COVID-19 cases in Portugal (from March 2) and the Republic of Korea (from February 15) by April 19, 2020.

	**Parameter**	**Estimate**	**Standard error**	***t*-value**	***p*-value**
**Portugal**
	*b*	0.60	1.53E-01	3.9315	0.0002885
	*c*	51.52	4.76E+01	1.0822	0.2849116
	*d*^a^	21,559.00	1.45E+03	14.9038	<2.2E-16
	*E*	69.50	1.30E+01	5.3548	2.799E-06
	*F*	2.40	3.98E-01	6.0222	2.894E-07
**Republic of Korea**
	*b*	0.48	1.49E-02	32.185	<2E-16
	*c*	−66.75	1.33E+02	−0.502	0.6176
	*d*^a^	7,306.07	1.25E+02	58.253	<2E-16
	*E*	27.69	1.92E-01	144.09	<2E-16
	*F*	1.71	9.64E-02	17.706	<2E-16

a *Parameter d is the predicted maximum*.

**Figure 2:**
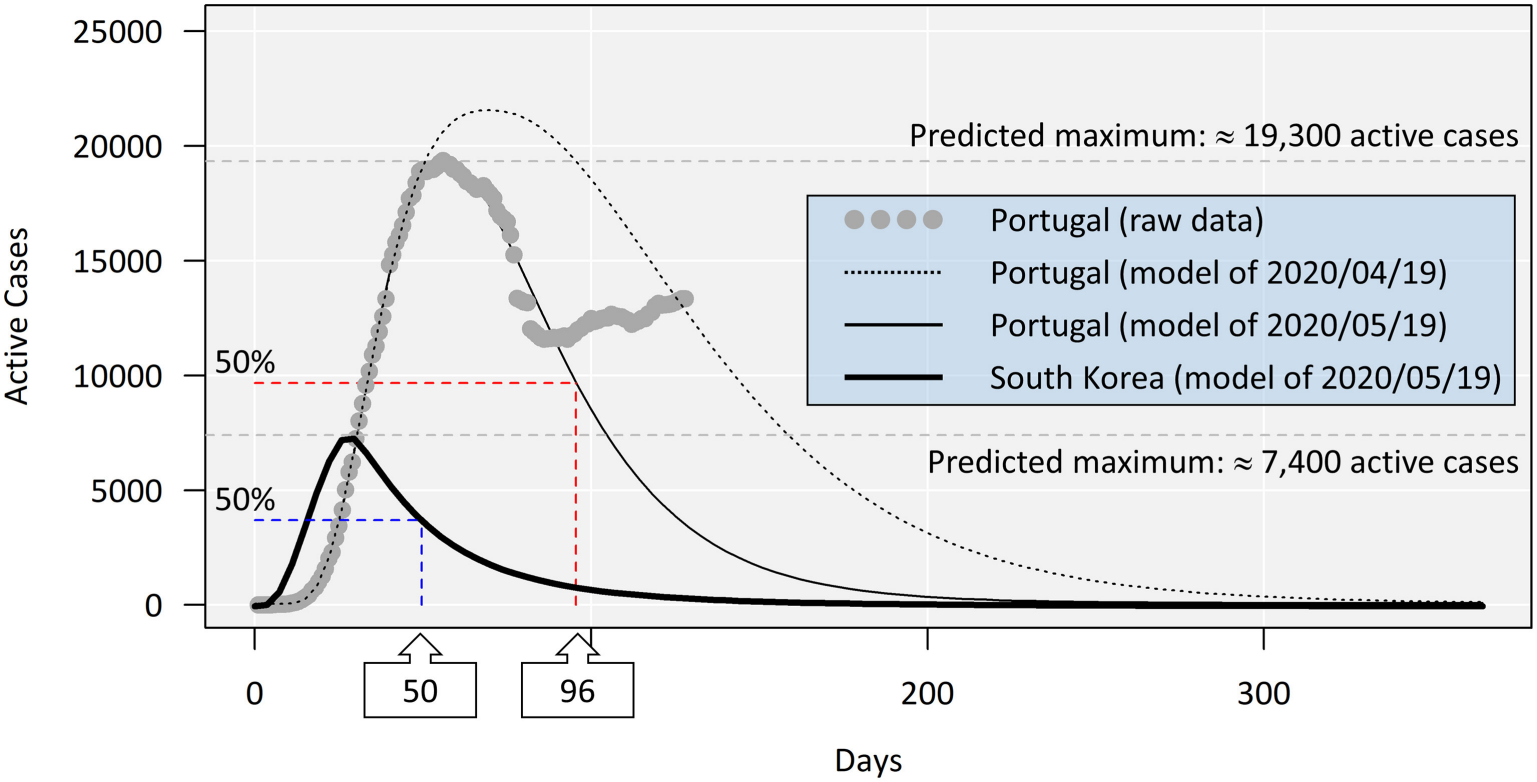
Comparison between models with updated COVID-19 active case data as of May 19, 2020. The models were produced as before (refer to Figure 1E). The two models for Portuguese data are exactly 1 month apart and show a more positive panorama predicted by the model fitted to data from May 19. The actual observations (consisting of war data up to July 8) are juxtaposed for comparison, highlighting the formation of a secondary peak after the loosening of lockdown measures (after May 20), compromising all predictions from May 19 onward. As the two models for the South Korean data overlap entirely, only the most recent is shown.

We then produced models with updated data from March 2 to May 19. This date marks not only a full month from the preceding model but also the début of a surge in new cases in Portugal after the easing of lockdown measures (Figure 2). This new model that accommodates data until the new surge now covered the peak in active cases and the descending curve, yielding much more promising results and factually showing a reduction in the half-maximal number of infected patients to day 96, comparatively to the previous model (day 140). In turn, the updated model of Korean cases overlapped the preceding. From May 19 to present (July 8), the number of active cases in Portugal not only interrupted its downward trend but actually increased, disrupting the expected biphasic model to reveal a clear secondary peak. This occurrence hinders further attempts to fit any standard models, rendering it difficult to predict COVID-19 clearance from the population. However, log-logistic regression using the data up to July 8 provides an estimate of 56,100 total infections (±2,600), which, compared with the 25,500 estimated prior to the end of lockdown mentioned above, represents 220% increase. The new maximum is, however, biased by uncertainty and is likely to increase over time with or without further outbreaks.

The new surges in Portugal, mostly located in the urban and the suburban areas of Lisbon, remain yet to be explained; however, they are seemingly a consequence of the easing of lockdown, despite persisting norms, e.g., on the use of public transportation at reduced capacity or limiting the number of attendees in public gatherings. Other emerging hotspots in further regions are also raising concerns as they affect both the elderly and the active portion of the population (with an increase in infants and adolescents as well). It must be noted, though, that the effect of age structure in the infected population under lockdown is still under debate. While the subject has hardly been analyzed in Portugal, there are indications from neighboring Spain that the posterior phases of a lockdown led to an increased proportion of infected adolescents and young adults (12), which may be mirrored in Portugal during the phased easing of the confinement measures. It must be noted that the increase in active cases in Portugal seems to be in line with reports elsewhere after the end of lockdowns, including in Europe. An example of this is the current situation in Barcelona and Galicia, both in Spain, which led to a new series of confinement measures. Comparisons in Europe are, however, difficult due to the lack of fully curated data and inconsistencies in the type and the rate of data release (e.g., absent data on recovered patients, which is needed to determine the real number of active cases), plus the differential effort to promote testing.

**Figure 3:**
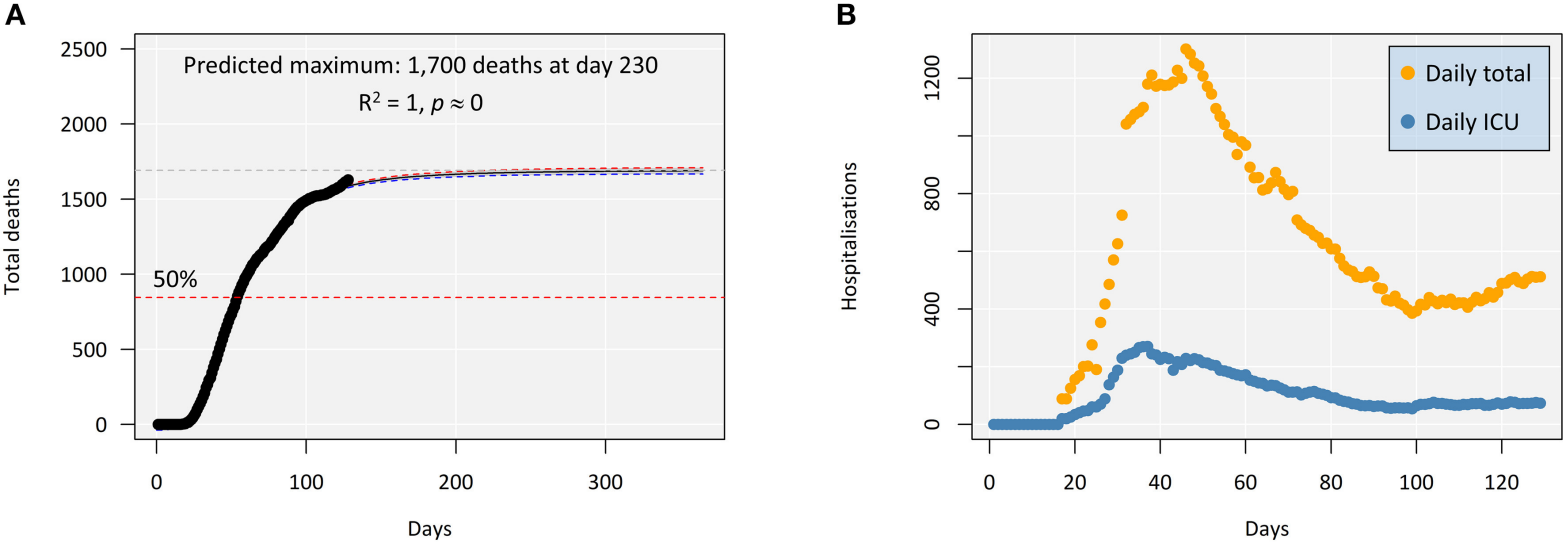
Indicators of COVID-19 progression in Portugal as of July 8, 2020. **(A)** Evolution of mortality, plotted for a full year to better highlight the asymptotic maximum. The shaded areas between the red and the blue dashed lines indicate 95% confidence intervals around the predicted model. **(B)** Daily hospitalizations in Portugal, total and in intensive care units. Day “zero” corresponds to March 2, the acknowledged beginning of the epidemic in Portugal.

The findings, which represent a rollback from the promising outcomes before the easing of the confinement rules in Portugal, can be further attested by a new predicted mortality maximum, ca. 1,700 against ca. 1,000 casualties that we described earlier (Figure 3A). In addition, the number of total daily hospitalized cases (*i*. *e*., new admittances minus discharged patients) is slowly but steadily rising again (Figure 3B), increasing linearly between June 6 and July 6 (*R*^2^ = 0.89, *p* ≈ 0) at a rate of four new cases per day. A similar trend is observed in ICU admittances. In addition to these new figures, novel findings disclosing reduced-immunity areas in Spain, where massive testing for seropositive individuals was conducted, even in hotspot areas (13), indicate that we should not expect significant group (“herd”) immunity. To this risk, we must add the fact that the persistence of the virus increases the odds of mutation. Regardless of the discussion on whether or not lockdown, partial lockdown, or even intermittent lockdown are actually effective [see, for instance, (14)], the situation in Portugal shows a cleavage between lockdown and post-lockdown. It must be noted, though, that albeit between-countries variability is likely, cross-national studies have highlighted that longer lockdown periods may effectively decrease dissemination and lead to fewer infected people [see (15)]. Considering the number of active cases in Portugal by the end of April, there is every possibility that the duration of the lockdown was a key factor.

## Conclusions

In countries such as Portugal, whose economy is heavily reliant on tourism and retail, post-lockdown outbreaks offer particular complications for the control and the mitigation of the epidemic and compromise predictive modeling. Our findings suggest that the current surge in active cases in Portugal may be, in part, due to the relatively high number of infected subjects before the easing of the lockdown measures as these figures are likely to reflect a very significant percentage of asymptomatic, unreferenced cases. Regardless of the causes for the new outbreaks, the present exercise is a contribution to highlight that predicting the dissemination of the new coronavirus is complex and hindered by new surges. These result in a much higher number of infections than predicted before the easing of the lockdown. In Portugal alone, the updated figures represent more than 200% increase in infected people than projected earlier, a difference that will likely grow in the forthcoming months.

## Data Availability Statement

The datasets presented in this study can be found in online repositories. The names of the repository/repositories and accession number(s) can be found in the article/Supplementary Material.

## Author Contributions

AM: data collection, interpretation of results, and manuscript writing. PC: statistical modeling and computation, interpretation of results, and manuscript writing. Both authors contributed to the article and approved the submitted version.

## Conflict of Interest

The authors declare that the research was conducted in the absence of any commercial or financial relationships that could be construed as a potential conflict of interest.
